# Family resilience and childhood obesity among children exposed to adverse childhood experiences in a national survey

**DOI:** 10.1002/osp4.497

**Published:** 2021-12-08

**Authors:** William J. Heerman, Lauren R. Samuels, Tavia González Peña, Chelsea van Wyk, Lindsay S. Mayberry, Julie Lounds Taylor, Nina C. Martin

**Affiliations:** ^1^ Department of Pediatrics Vanderbilt University Medical Center Nashville Tennessee USA; ^2^ Department of Biostatistics Vanderbilt University Medical Center Nashville Tennessee USA; ^3^ Vanderbilt University School of Medicine Nashville Tennessee USA; ^4^ Department of Medicine Vanderbilt University Medical Center Nashville Tennessee USA; ^5^ Vanderbilt Kennedy Center Vanderbilt University Medical Center Nashville Tennessee USA; ^6^ Peabody College of Education Vanderbilt University Nashville Tennessee USA

**Keywords:** adverse childhood experiences, childhood obesity, family resilience

## Abstract

**Objective:**

Adverse childhood experiences (ACEs) contribute to poor overall health among children with obesity. This study evaluated how one potential protective factor—family resilience—affects the association between ACEs and childhood obesity.

**Methods:**

This analysis was a secondary analysis of the 2016–2018 National Survey of Children's Health (NSCH), a repeated cross‐sectional survey based on parent report. Nine ACEs were queried. Family resilience was assessed with four items (potential range 0–12). The primary outcome was child weight status. Multivariable ordinal logistic regression was used, adjusting for potential confounders and the interaction between ACEs and family resilience.

**Results:**

For 49,365 children ages 10–17, the median number of ACEs was 1 (IQR 0, 2), the median family resilience score was 10 (IQR 8,12), 15.3% of children had overweight, and 15.4% of children had obesity. Among the 51.3% of children who experienced one or more ACEs, higher family resilience scores attenuated the odds of being in a higher weight category. This pattern was not observed in children with zero ACEs.

**Conclusions:**

In the 2016–2018 NSCH, children ages 10–17 who were exposed to ACEs had higher rates of overweight and obesity, the odds of which may be reduced when children also have higher family resilience.

## 1 INTRODUCTION

The disproportionate prevalence of childhood obesity among low‐income and racial/ethnic minority populations continues to be a major health inequity in the United States.^1^ There are multiple contributing causes to this inequity, including higher rates of social trauma.^2^ Adverse childhood experiences (ACEs) are associated with higher rates of childhood obesity and other long‐term health risks.^3^, ^4^ In addition, social disadvantage and a corresponding lack of protective resources exacerbate the risk of poor health related to social trauma.^5^, ^6^ However, social trauma does not deterministically lead to poor health outcomes. Protective factors like positive maternal mental health, neighborhood and school safety, and child resilience can reduce the risk of childhood obesity even in the face of ACEs.^7^ Thus, characterizing protective factors that interrupt the pathway from social trauma to childhood obesity could augment intervention strategies designed to reduce health disparities among children exposed to ACEs.

ACEs are linked with childhood obesity through a well‐established pathway. ACEs are childhood social traumas caused by abuse, neglect, violence, substance abuse, parent separation, peer victimization, community violence, unemployment, and property victimization.^8^, ^9^ These social traumas incite a physiological stress response that when experienced repeatedly, becomes toxic, altering the neural circuitry controlling these neuroendocrine (i.e., stress) responses.^10^, ^11^, ^12^ The stress response has a complex and reciprocal relationship with healthy behaviors (e.g., healthy diet patterns, physical activity, sleep). Healthy behaviors can reset a dysregulated stress response, while stress‐associated social‐emotional dysfunction (e.g., poor self‐regulation) can reduce the capacity to engage in healthy behaviors. This reduced capacity perpetuates or even heightens the stress response and results in negative health outcomes such as childhood obesity.^10^, ^13^

Resilience is an adaptation to adversity that “transforms potentially toxic stress into tolerable stress.”^14^ Specifically, family resilience “is the successful coping of family members under adversity that enables them to flourish with warmth, support, and cohesion.”^15^ Conceptually, family resilience is one protective factor that may be especially important for reducing the impact of ACEs on child obesity.^16^, ^17^ The hypothesized mechanisms by which family resilience potentially mitigates the impact of ACEs on childhood obesity are through the provision of (1) loving and safe parent–child relationships that reduce the impact of toxic stress and (2) stable routines (e.g., family mealtimes, quality time via family activities) that facilitate engagement in health behaviors to promote health child growth patterns. In addition, family resilience is not static, but rather something that can be promoted by targeted intervention.^18^

Despite a strong conceptual relationship, associations between family resilience and childhood obesity have not been consistently reported. Notably, a recent investigation using the 2016 National Survey of Children's Health (NSCH) did not find associations between family‐level resilience and lower rates of childhood obesity.^16^ One potential explanation is that family‐level resilience may only be activated as a protective factor for childhood obesity in the context of stressors, in this case ACEs. It may also be that family resilience may have different effects on health outcomes in some population subgroups.^19^ ^,^ ^20^ ^,^ ^21^ With the goal of reducing health disparities in childhood obesity by targeting socially protective factors among traditionally minoritized communities, a careful examination of how resilience functions in communities of color should be a priority.

The purpose of this study was to examine how family resilience affects the association between ACEs and childhood overweight and obesity in a nationally representative sample of children ages 10–17. A second purpose of this study was to examine the extent to which the relationships between ACEs, family resilience, and overweight and obesity may vary by race and ethnicity. We hypothesized that children with higher family resilience scores would have an attenuated association between ACEs and childhood overweight and obesity when compared to children with lower family resilience scores. We also hypothesized that the relative strength of this attenuation would vary by a child's race or ethnicity.

## 2 METHODS

We conducted a secondary analysis of data collected via the NSCH, a repeated cross‐sectional survey of households in the United States with at least one child.^22^ The purpose of NSCH is to collect data on the physical and emotional health of children 0–17 years old in the United States. The survey has been administered six times since 2003 and conducted annually by the US Census Bureau since 2016. Our analysis combines sequential data from 2016–2018, following published guidance for combining the data sets. All data from the publicly available data set are nonidentifiable. The Institutional Review Board of Vanderbilt University Medical Center, Nashville, Tennessee, approved the current analysis plan.

The NSCH 2016–2018 survey administration methods are publicly available.^22^ The survey was conducted in both English and Spanish and administered online and by mail. The US Census Bureau initially identified households that were likely to have a child living in the home, stratified by state. Households were then screened to confirm eligibility, and one child per household was randomly selected as the subject of the survey. The weighted overall response rate in 2016 was 53.0%, in 2017 was 37.4%, and in 2018 was 40.7%. The respondent was a parent or caregiver in the home who could answer questions about the child's health. The sampling strategy was designed to over‐sample children with special healthcare needs. To obtain population‐based estimates, each child for whom a survey was completed was assigned a weight that included adjustments for the base sampling weight, non‐response, the selection of single child in a household, and demographic characteristics.

The primary independent variable for this analysis is a count of ACEs experienced by the child and ranges from 0 to 9. The survey included nine items to assess a child's exposure to the following ACEs: (1) difficulty covering basic needs like food or housing, (2) parent divorce or separation, (3) death of a parent, (4) parent jail time, (5) domestic violence, (6) neighborhood violence, (7) household mental illness, (8) household drug or alcohol abuse, and (9) being treated unfairly because of race or ethnic group. The NSCH calculates a total count of ACEs for children who have at least one response to the nine ACEs items, with any missing values coded as “not exposed.”^23^

The primary outcome for this analysis is child weight status based on parent‐reported height and weight, categorized using standardized cut‐points from the CDC.^24^ Four categories were designated: underweight (BMI less than the 5th percentile), normal weight (5th to 84th percentile), overweight (85th to 94th percentile), or obesity (95th percentile or above). Neither raw height and weight nor the NSCH‐calculated BMI are reported in the NSCH data sets, precluding the use of a continuous weight‐related outcome measure. For the current analysis, children who were underweight were combined with children who were normal weight to create an ordinal outcome variable with three possible values: underweight/normal weight, overweight, and obesity.

Family resilience was assessed with four survey items: “When your family faces problems, how often are you likely to do each of the following?” (1) talk together about what to do, (2) work together to solve problems, (3) know we have strengths to draw on, and (4) stay hopeful even in difficult times. Responses options were “none of the time,” “some of the time,” “most of the time,” or “all of the time.” We coded these responses on a 4‐point Likert scale from 0—“none of the time” to 3—“all of the time.” Responses were summed to create a continuous measure of family resilience with a possible range of 0–12, where higher numbers correspond to higher family resilience. We calculated this summary score only for participants with complete data on family resilience, excluding children who were missing any items on the family resilience scale. The weighted Cronbach's alpha for this scale was 0.89.

To account for potential confounding, we adjusted for variables that were hypothesized to be associated with both the exposure and the outcome but not part of the causal pathway. We conceptualized potential confounders using a social‐ecological framework, recognizing the potential influence of individual, family, and community level factors. Each of the covariates was coded as suggested in the NSCH Codebook.^23^ At the individual level, we controlled for child age, child sex (male; female), child race/ethnicity (Hispanic; Black, non‐Hispanic; white, non‐Hispanic; other, non‐Hispanic), and current health insurance status (yes; no). Recognizing the potential contribution of chronic health conditions, we also included a variable indicating whether a child had special healthcare needs (yes; no). At the family level, we controlled for highest educational level of an adult in the household (less than high school; high school or GED; some college or technical school; college degree or higher), the family structure (two parents, currently married; two parents, not currently married; single mother; other family type), and total number of people living in the household (2; 3; 4; 5; ≥6). We did not control for family income level, as it is assessed in the first item in the ACEs scale. At the community level, we controlled for whether the neighborhood was supportive, using an NSCH‐derived dichotomous measure (yes; no) derived from three items. We also controlled for the number of available neighborhood amenities like sidewalks, parks, community centers, and so on. (0; 1; 2; 3; 4).

### 2.1 Statistical analysis plan

All analyses, including descriptive analyses, were conducted using survey weights provided in the NSCH data set, following NSCH methodology for both combining surveys from multiple years and analyzing subsets of the data. For the primary outcome of child weight status, we first report the prevalence of child weight status by number of ACEs experienced and then by the combination of ACEs and family resilience scores. To evaluate how family resilience affected the association between exposure to ACEs and child weight status, we used an ordinal logistic regression (OLR) model with three terms for the number of ACEs (1, 2–3, 4–9; reference = 0), a single term for score on the family resilience scale, and three interaction terms between family resilience and number of ACEs, adjusting for the covariates described above. The categorization of ACEs into four groups was based on the distribution of responses across combinations of ACEs and family resilience scores (shown in Table S1 and Figure S1), in order to ensure adequate data density for modeling.

Because of the complexity of the OLR model, we used post‐estimation techniques to present results in two formats. First, we calculated odds ratios across the range of ACEs and family resilience scores. The odds compared in these odds ratios, as with all OLR models, are the odds of being in a higher versus lower outcome category under any possible dichotomization of the outcome. Our three‐level outcome has two possible dichotomizations: obesity versus not obesity; or obesity/overweight versus normal/underweight. The odds ratios we calculated give the relative odds of being in a higher versus lower weight category using either of these dichotomizations, compared to children with no ACEs and a family resilience score of 0. To obtain insight into the absolute probabilities of obesity or overweight predicted by the model, we then calculated predicted probabilities of a child having overweight or obesity across the range of ACEs exposure and family resilience scores. Unlike odds ratios, predicted probabilities from an OLR model depend on a child's covariate values. We calculated predicted probabilities for children from each race/ethnicity category, using the median age and the most common values for the other adjusting covariates.

To evaluate whether the relationship between family resilience, ACEs, and child weight status differed by race/ethnicity, we constructed an expanded model that also included three‐way interaction terms between a child's race/ethnicity, number of ACEs, and family resilience score, as well as the relevant lower‐order interactions. These lower‐order interactions allow the relationships between ACEs and weight status and between family resilience and weight status to vary by race/ethnicity.

### 2.2 Sensitivity analysis

Because missing answers to items on the ACE screener had different patterns of missingness depending on both the nature of the ACE and the child's race/ethnicity, we also conducted a sensitivity analysis in which we included only children with complete data on at least 6 ACEs and treated missing ACE items as “exposed” in calculating the total number of ACEs.

### 2.3 Missing data

NSCH used hot‐deck imputation for missing values in race, ethnicity, and sex; and regression‐based imputation techniques for household size and the highest education level of the primary adult.^23^ We then used multiple imputation via predictive mean matching to impute missing values for the remaining covariates in our model. All analyses were summarized over the 10 resulting data sets and conducted using R version 4.0.2.^25^

## 3 RESULTS

**FIGURE 1:**
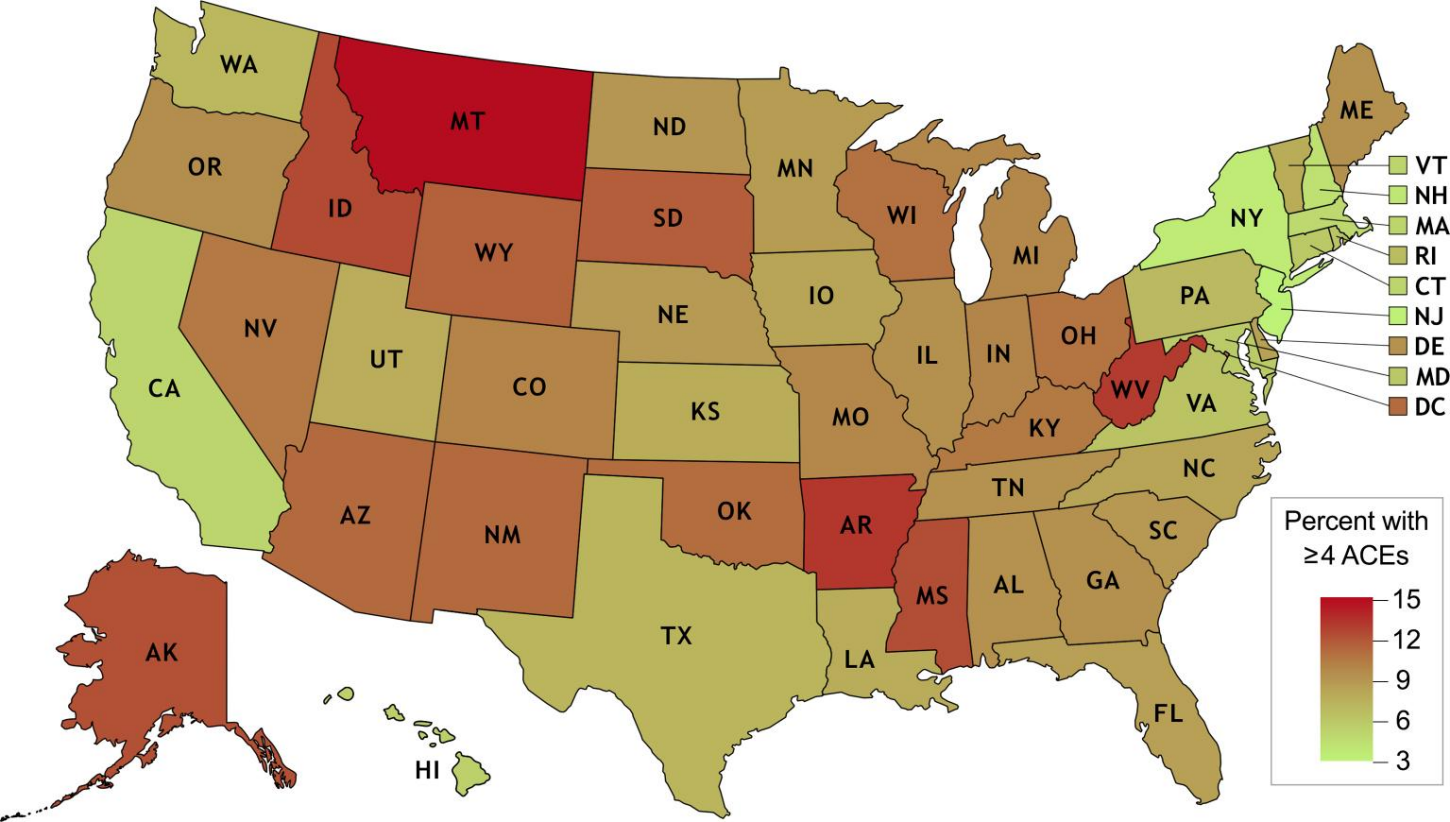
Percent of children who have experienced ≥4 adverse childhood experiences, by state

Of the 53,422 children ages 10–17 who were included the 2016–2018 NSCH, 49,365 (90.3%) were included in the present analysis. Children were excluded sequentially if they had missing data on all ACEs (*N* = 709), any of the items on the family resilience score (*N* = 993), or weight category (*N* = 2355). The final sample of 49,365 included children from each of the survey years of interest (2016: *N* = 23,823; 2017:10,511; 2018:15,031) and, with survey weighting, represents 30,023,428 children ages 10–17 in the United States. The demographic characteristics of the sample are presented in Table 1, both overall and stratified by exposure to ACEs. The median number of ACEs experienced by children in this sample was 1 (IQR 0, 2), with significant geographic variability (Figure 1). Approximately half of children reported no ACEs (48.68%), 25.48% reported 1 ACE, 11.82% reported 2 ACEs, 6.04% reported 3 ACEs, and 7.96% reported ≥4 ACEs. The median score on the family resilience scale was 10 (IQR 8,12). The majority of children were reported to have normal weight or underweight (69.3%), with 15.3% of children having overweight, and 15.4% of children having obesity.

**TABLE 1:** Demographic characteristics, overall, and by categorized ACE count

	Overall	0 ACEs	1–3 ACEs	4–9 ACEs	*p*	% Missing
*N* (weighted sample size)	30,023,428	14,615,973	13,015,195	2,392,259		
Child age (median [IQR])	14 [12, 16]	13 [11, 15]	14 [12, 16]	14 [12, 16]	<0.001	0
Child sex (%)					0.778	0.1^a^
Male	51	50.6	51.3	51.7		
Female	49	49.4	48.7	48.3		
Child race/ethnicity (%)					<0.001	1.3^a^
Hispanic	24.8	24.9	25.0	22.9		
White, non‐Hispanic	52.0	56.1	48.5	46.1		
Black, non‐Hispanic	13.5	8.9	17.6	19.7		
Other/Multi‐racial, non‐Hispanic	9.7	10.2	8.9	11.3		
Child has current insurance coverage (%)	93.3	93.6	93	93.8	0.556	0.2^b^
Child has special healthcare needs (%)	23.9	17.6	27.2	44.5	<0.001	0
Adult highest education level (%)					<0.001	1.3^a^
Less than high school	10.7	10.8	10.6	11.4		
High school or GED	19.5	14.2	23.8	28.6		
Some college or technical school	23.1	17.7	27.2	33.6		
College degree or higher	46.7	57.3	38.4	26.3		
Family structure (%)					<0.001	0.4^b^
Two parents, currently married	65.3	87.9	47.7	23.1		
Two parents, not currently married	7.4	2.8	12.3	8.7		
Single mother	18.0	5.6	27.5	40.7		
Other family type	9.4	3.6	12.5	27.5		
Number of people in household (%)						
Two people	4.9	1.1	8.3	10.2	<0.001	1.0^a^
Three people	19.6	15.9	23.3	22.5		
Four people	32.5	38.1	28.3	20.8		
Five people	25.1	27.8	22.2	24.4		
Six or more people	17.9	17.1	17.9	22.1		
Family lives in supportive neighborhood (%)	56.7	66.7	49.2	35.8	<0.001	1.4^b^
Neighborhood amenities (%)					<0.001	1.8^b^
Neighborhood does not contain any amenities	11.3	10.2	11.8	15.6		
Neighborhood contains one amenity	10.9	10.4	11.2	12.5		
Neighborhood contains two amenities	16.2	14.7	17.6	18		
Neighborhood contains three amenities	22.8	21.9	23.6	23.5		
Neighborhood contains all four amenities	38.8	42.8	35.8	30.5		

*Note*: All data are presented using survey weights.

^a^ Imputed by NSCH; imputed values are included in the table

^b^ Imputed by study team; imputed values are not included in the table

**FIGURE 2:**
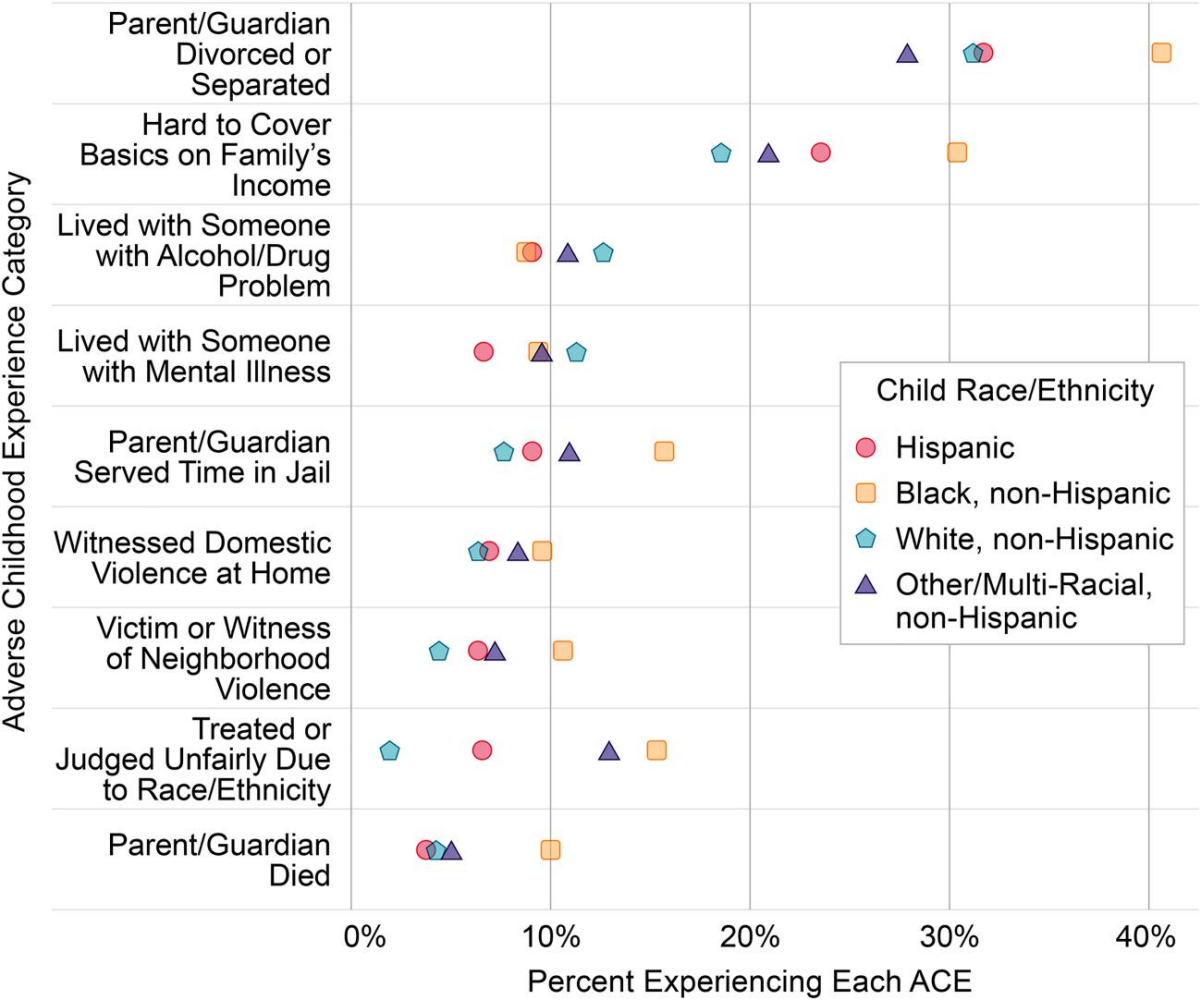
Percent of children experiencing each category of adverse childhood experience by race/ethnicity

Significant differences existed in the distribution of ACEs and obesity by child race/ethnicity. Children who were Hispanic or Black, non‐Hispanic experienced a higher total number of ACEs compared to White, non‐Hispanic children. A higher proportion of Black, non‐Hispanic children experienced individual ACEs across seven of the nine domains (Figure 2). The distribution of childhood overweight and obesity also differed by child race/ethnicity. Overall, 69.3% of children were normal or underweight, compared to 74.1% of white, non‐Hispanic children, 62.3% of Hispanic children, and 61.4% of Black, non‐Hispanic children. Conversely, 12.2% of white, non‐Hispanic children had obesity, compared to 19.6% of Hispanic children and 22.2% of Black, non‐Hispanic children. In contrast, only minor differences existed in family resilience score by race/ethnicity. The full distribution of ACEs, family resilience score, and weight status overall and by race/ethnicity are shown in Tables S2 and S3. Importantly, children with special healthcare needs experienced higher rates of each ACE category compared to children without special healthcare needs (Table S4 and Figure S3).

Children who experienced higher numbers of ACEs also had higher prevalence of childhood overweight and obesity. Prevalence of overweight ranged from 14% in children unexposed to ACEs to 18% in children exposed to 4–9 ACEs, while prevalence of obesity ranged from 11% to 21% in those groups, respectively (Table 2). The prevalence of child overweight and obesity is shown for specific combinations of ACEs and family resilience in Table 3.

**TABLE 2:** Prevalence of overweight and obesity by number of ACEs

Number of ACEs	Normal/Underweight (%)	Overweight (%)	Obesity (%)
0	74	14	11
1	67	16	18
2–3	63	16	21
4–9	61	18	21

Abbreviation: ACE, adverse childhood experience.

**TABLE 3:** Prevalence of overweight and obesity for combinations of ACEs and Family Resilience scores

Number of ACEs	Family Resilience 0		Family Resilience 4		Family Resilience 8		Family Resilience 12	
	Overweight (%)	Obesity (%)	Overweight (%)	Obesity (%)	Overweight (%)	Obesity (%)	Overweight (%)	Obesity (%)
0	22	4	18	11	13	10	16	12
1	22	10	15	24	15	15	16	19
2–3	18	5	20	22	17	21	17	21
4–9	21	35	15	18	21	17	17	23

*Note*: Percentages represent the survey‐weighted prevalence estimates for child overweight and obesity for specific combinations of ACEs and Family Resilience Score (possible range 0–12).

Abbreviation: ACE, adverse childhood experience.

**FIGURE 3:**
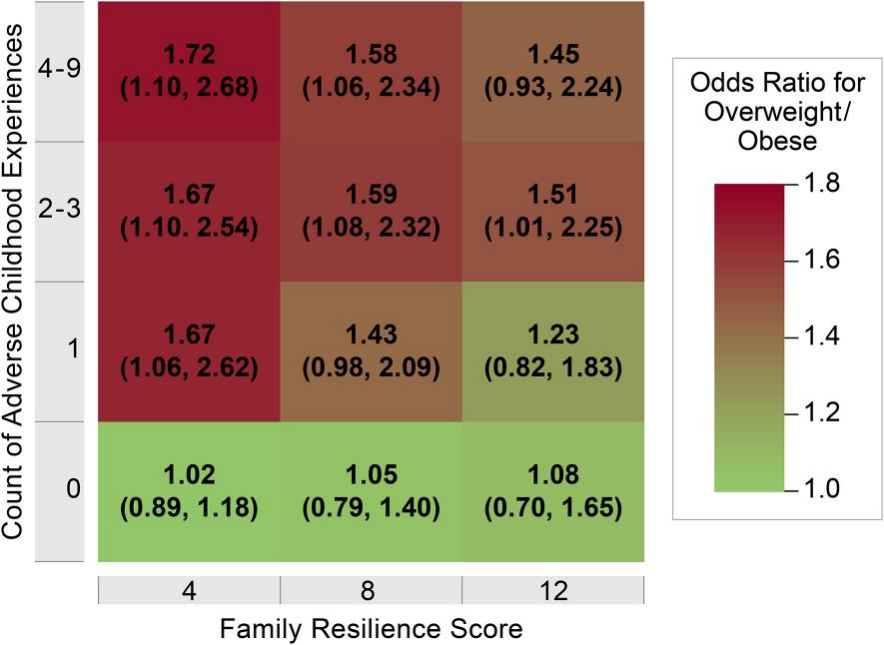
Family resilience, adverse childhood experiences, and odds ratios for overweight/obesity. Odds ratios from the adjusted ordinal logistic regression model are shown for specific combinations of adverse childhood experience (ACE) count and family resilience score. Odds ratios are with respect to the reference categories of 0 ACEs and a family resilience score of 0. For example, children with a family resilience score of 4 and 4–9 ACEs have an odds of being in a higher versus lower weight category that is 1.72 times the odds for children with zero ACEs and a family resilience score of 0. “Being in a higher versus lower weight category” applies to either of the two possible dichotomizations: obesity versus overweight/normal/underweight, or obesity/overweight versus normal/underweight. Odds ratios in the figure are shown with 95% confidence intervals. The odds ratios reported are derived from combinations of odds ratios from the overall adjusted ordinal logistic regression model; the full output of which is shown in Table S5

**FIGURE 4:**
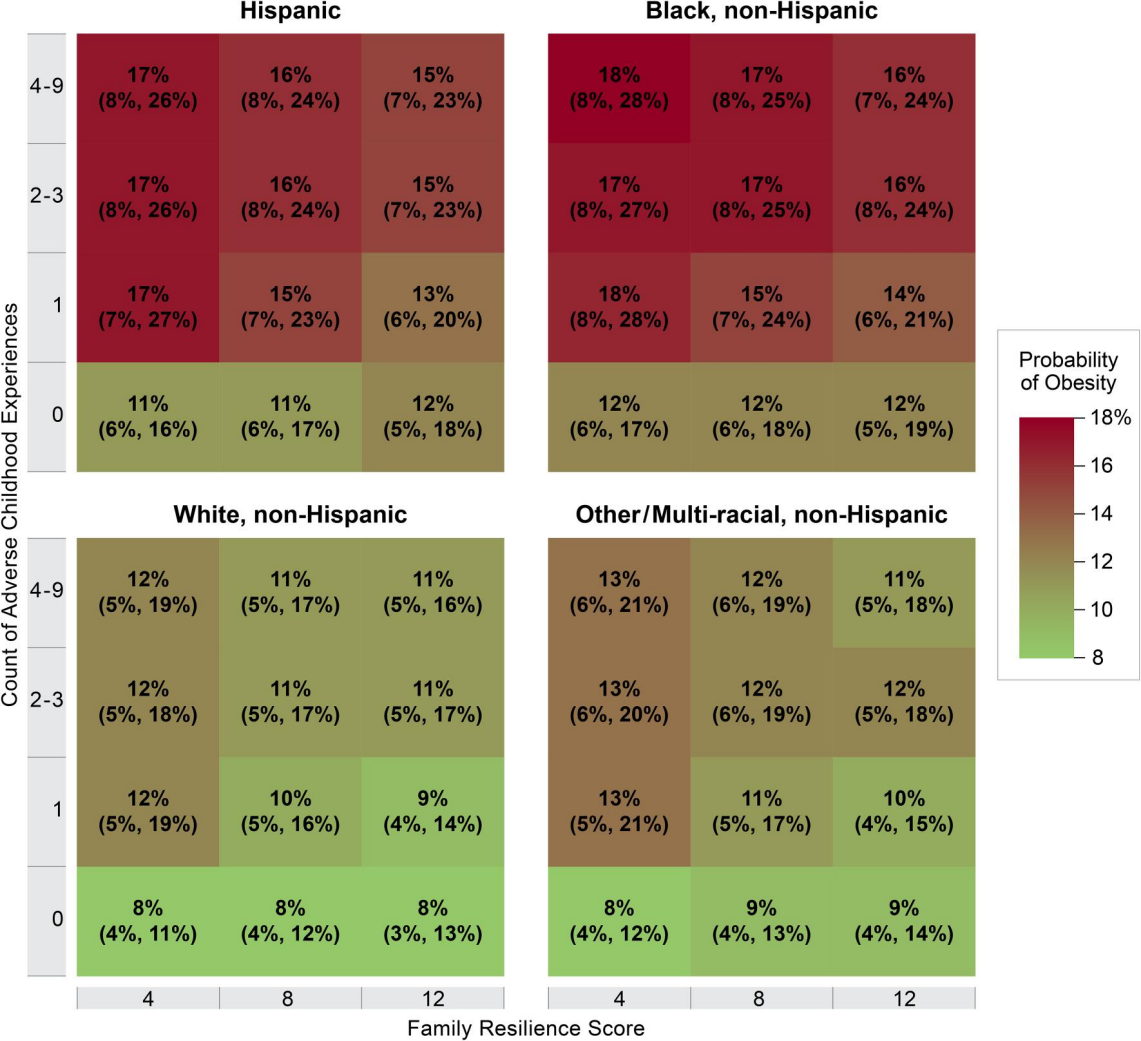
Predicted probabilities of child obesity by race and ethnicity. The predicted probability of child obesity is shown for specific combinations of adverse childhood experience count and family resilience, based on the adjusted proportional odds regression model. The predicted probability is shown with the 95% confidence interval. To obtain these probabilities, covariate profiles were set to the specified race/ethnicity, with other covariates set to the median or mode. The predicted probabilities are derived from the overall adjusted ordinal logistic regression model, the full output of which is shown in Table S5

As noted above, we present two model‐based summaries of the results from the adjusted OLR model. Figure 3 shows that among children who experienced ACEs, higher family resilience scores were associated with lower odds of being in a higher weight category. The converse was also observed: family resilience did not have the same attenuating effect on the odds of childhood overweight and obesity among children who were not exposed to ACEs. Figure 4 shows that among children with typical covariate profiles, the predicted probabilities of obesity were higher among Hispanic and Black, non‐Hispanic children at all combinations of ACEs and family resilience. The full results from the main model are presented in Table S5. The results of the additional model that adds three‐way interaction terms failed to detect a race/ethnicity‐based difference in the manner in which family resilience decreased the odds of a higher weight category at any level of ACEs (data not shown). In our sensitivity analysis, changing how missing ACE items were coded did not result in any substantive changes to the results (results not shown).

## 4 DISCUSSION

In the 2016–2018 NSCH, children aged 10–17 who were exposed to ACEs had higher rates of childhood overweight and obesity, the odds of which were attenuated when children also had higher family resilience. Despite the large sample size and nationally representative sampling strategy, the confidence intervals for specific combinations of ACEs and family resilience scores remained wide. This limits the ability of this analysis to firmly conclude that family resilience mitigates the effects of ACEs on childhood overweight/obesity. However, there is a consistent trend towards a “dose response,” whereby (1) children with more ACEs tend to have higher odds of overweight and obesity, and (2) among children with ACEs, children with higher scores on the family resilience scale have the most attenuation of the odds ratios between ACEs and overweight/obesity. It is also important to acknowledge that family resilience did not have the same attenuating effect on the odds of childhood overweight and obesity among children who were not exposed to ACEs. This observation supports the proposed mechanism by which family resilience may ameliorate the physical health consequences of ACEs, as we would not expect children who did not experience the social trauma of ACEs to have need of family resilience to buffer the toxic stress response. Taken together, these results indicate the need for further study into how family resilience might buffer a toxic stress response. We cannot yet firmly suggest intervention development in this area based on the lack of precision in the effect estimates (i.e., wide confidence intervals). However, the confidence intervals do include what would be a clinically meaningful reduction in the probability of overweight or obesity, suggesting that family resilience may be protective for children exposed to ACEs.

In this sample of 10–17‐year‐old children, more than half of children had been exposed to ≥1 ACE and nearly 8% had been exposed to ≥4 ACEs. The incredibly high rates of exposure to ACEs in this nationally representative sample indicate the pressing need for strategies to ameliorate the long‐term health effects of these social traumas in childhood. This study suggests that increasing family resilience may be one such strategy. Resilience protects children from social trauma and can be conceptualized at both the individual and family levels.^15^, ^17^, ^18^ A recent study conducted using data from the 2016 NSCH by Foster and Weinstein documented the association between child‐level components of resilience with lower prevalence of childhood obesity, but did not find an association between family‐level resilience and lower rates of childhood obesity.^16^ The current analysis used a similar conceptual framework as this earlier study but yielded the expected associations, perhaps in part due to the use of a larger sample size by compiling 3 years of NSCH data, the use of an ordinal outcome variable that did not aggregate childhood overweight and obesity into a single outcome, and the use of a continuous family resilience variable.

The data from this study also indicate important differences by race and ethnicity in the prevalence of ACEs and obesity, but not in how family resilience impacts the relationships between ACEs and obesity. Consistent with previous studies, data from the 2016–2018 NSCH indicate that Hispanic and Black, non‐Hispanic children have higher exposure to ACEs and higher prevalence of childhood overweight and obesity compared to white, non‐Hispanic children.^2^, ^26^ Importantly, the family resilience scores do not differ appreciably by race or ethnicity. Furthermore, we did not detect a difference by child race and ethnicity in the effect of family resilience on the association between ACEs and child weight status (i.e., the statistical interaction was not significant). Taken together, this suggests that bolstering family resilience regardless of a child's race/ethnicity may be one strategy to mitigate the health consequences of ACEs.

While not specifically an objective of this study, this analysis also identified significant differences in the rates of ACEs and obesity among children with special healthcare needs. This may indicate a unique population that requires careful attention in future studies, both to better characterize how ACEs are related to physical and mental health outcomes and to develop unique intervention strategies to address the social trauma to which these most vulnerable children are exposed.

This study had several limitations. First, the analysis was cross‐sectional, limiting the ability to draw causal inference or identify temporal relationships that could help elucidate potential mechanisms by which family resilience may mitigate the risk of ACEs on higher child weight status. Second, all the data were collected by parent report, which raises the possibility of misclassification of both the exposures and the outcome. This bias is likely differential, meaning parents may have been more likely to over‐ or under‐report child height and weight in a way that could bias the results in an unpredictable way. Third, because child BMI is calculated from the height and weight reported by parents, the NSCH elects to only provide categorical measures of child weight status, and not raw BMI. This inherent limitation of the data limits a more nuanced understanding of the impact of ACEs on child weight status. Fourth, as with any observational study, there is potential for residual confounding, which could potentially explain some of the observed associations. Fifth, the measure of family resilience in the current study had a distribution with a “ceiling effect,” such that there may have been more variability in family resilience than was captured by the current measure. Furthermore, the measure of family resilience selected by the NSCH may not address all potentially relevant domains, such as the stability of family routines. This may have led to misclassification of the exposure in this study. In addition, there was low data density at high ACEs values or very low family resilience, limiting the ability to draw inference about those specific combinations of values. This suggests an area of future study to develop additional measures of family resilience that could more precisely measure that latent construct.

## 5 CONCLUSION

In the 2016–2018 NSCH, children ages 10–17 who were exposed to ACEs had higher rates of childhood overweight and obesity, the odds of which were attenuated when children also had higher family resilience.

## CONFLICT OF INTEREST

The authors declare no conflict of interest.

## AUTHOR CONTRIBUTIONS

William J. Heerman formulated the study question, interpreted results, and drafted the initial manuscript. Lauren R. Samuels conducted the statistical analysis and created data representations. Tavia G. Peña, Chelsea van Wyk, Lindsay S. Mayberry, Julie L. Taylor, and Nina C. Martin all contributed to refining the study question, conceptualizing the analysis plan, interpreting results, and situating the current study within the existing literature. All authors contributed to critical revisions of the manuscript and approved the final version of the manuscript.

## Supporting information

Supporting Information S1
