# Supplementary material for: Family resilience and childhood obesity among children exposed to adverse childhood experiences in a national survey
Source: Obes Sci Pract. 2021 Dec 8;8(1):3–11. doi: 10.1002/osp4.497 (PMC8804940; doi:10.1002/osp4.497)
Supplement: Supplementary file 1 — Supporting Information S1 [file OSP4-8-3-s001.docx]

**Supplementary Material**

**Title: Family Resilience and Childhood Obesity Among Children Exposed to Adverse Childhood Experiences in a National Survey**

**Contact Author:** William Heerman, MD MPH. 2146 Belcourt Ave, 2^nd^ Floor. Nashville, TN 37212. [Bill.Heerman@vumc.org](mailto:Bill.Heerman@vumc.org)

**Distribution of Key Variables**

**Table S1: Distribution of counts of ACEs, overall**

| **Number of ACEs** | 0 | 1 | 2 | 3 | 4 | 5 | 6 | 7 | 8 | 9 |
| --- | --- | --- | --- | --- | --- | --- | --- | --- | --- | --- |
| **Percent of children** | 48.68 | 25.48 | 11.82 | 6.04 | 3.73 | 1.98 | 1.45 | 0.59 | 0.16 | 0.05 |

**Figure S1: Distribution of counts of ACEs, by Race/Ethnicity**

**
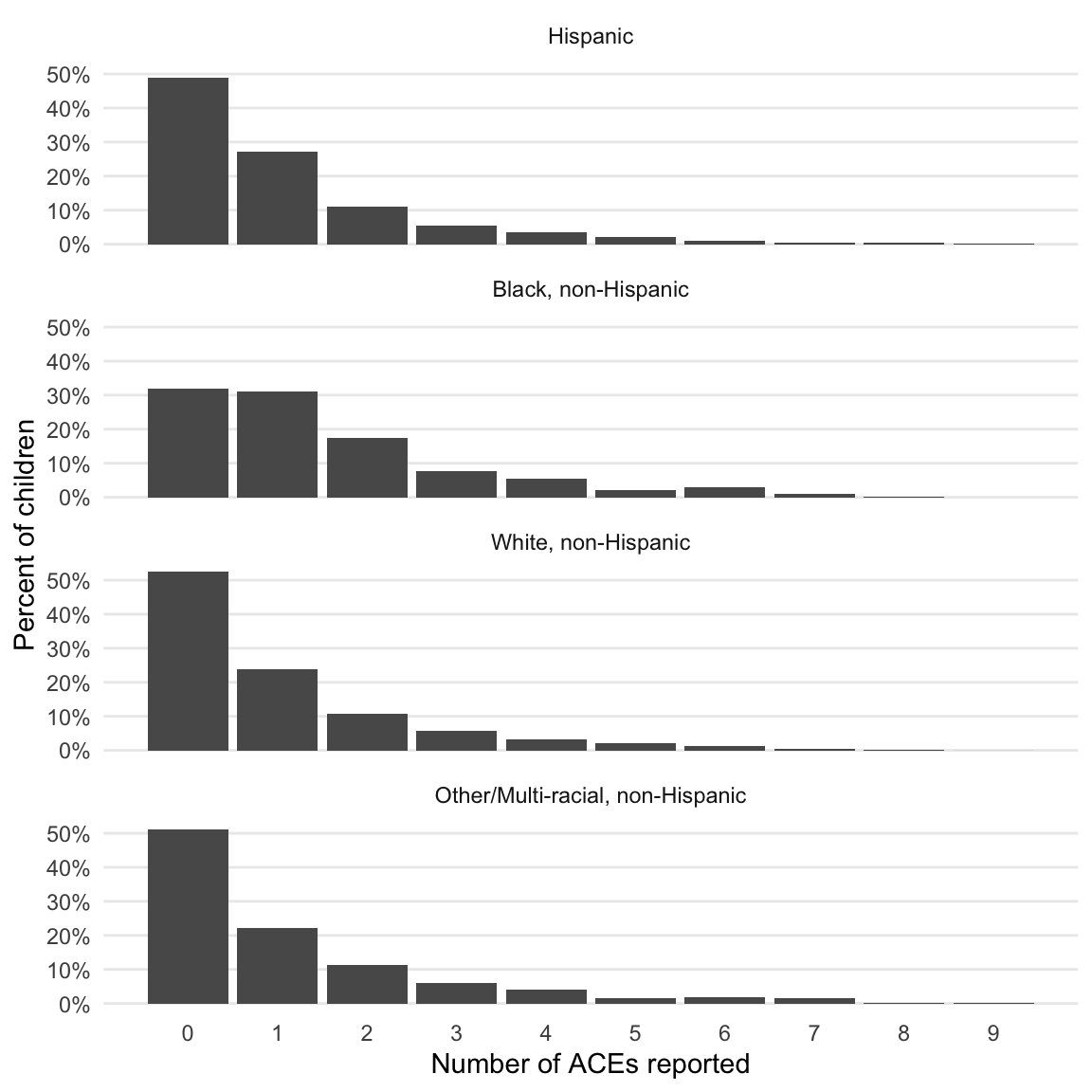
**

**Table S2: Number of ACEs, family resilience score, and weight status: Overall and by race/ethnicity category.**

|  |  | **Overall** | **Hispanic** | **White, non-Hispanic** | **Black, non-Hispanic** | **Other/ Multi-racial, non-Hispanic** |
| --- | --- | --- | --- | --- | --- | --- |
| **N**  (weighted sample size) | | 30,023,428 | 7,437,238 | 15,611,642 | 4,053,329 | 2,921,218 |
| **Number of ACEs** (median [IQR]) | | 1 [0, 2] | 1 [0, 1] | 0 [0, 1] | 1 [0, 2] | 0 [0, 2] |
| **Family resilience score** (median [IQR]) | | 10 [8, 12] | 10 [8, 12] | 9 [8, 12] | 10 [8, 12] | 9 [8, 12] |
| **Weight status** (%) | |  |  |  |  |  |
|  | Normal/under | 69.3 | 62.3 | 74.1 | 61.4 | 72.5 |
|  | Overweight | 15.3 | 18.1 | 13.7 | 16.4 | 15 |
|  | Obese | 15.4 | 19.6 | 12.2 | 22.2 | 12.5 |

**Table S3: Distribution of family resilience score:**

| **Family resilience score** | 0 | 1 | 2 | 3 | 4 | 5 | 6 | 7 | 8 | 9 | 10 | 11 | 12 |
| --- | --- | --- | --- | --- | --- | --- | --- | --- | --- | --- | --- | --- | --- |
| **Percent of children** | 0.34 | 0.36 | 0.32 | 0.64 | 3.28 | 3.11 | 4.29 | 4.59 | 21.93 | 9.36 | 10.07 | 8.38 | 33.33 |

**Figure S2: Joint distribution of ACE count & family resilience score:**


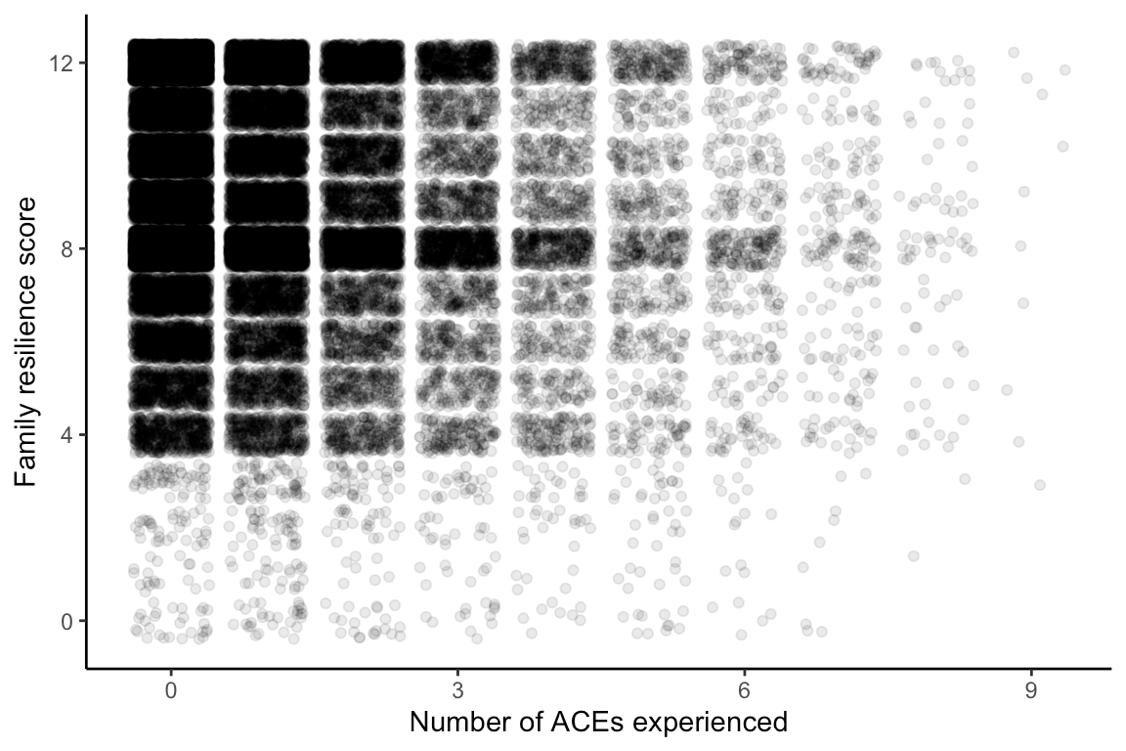


**Table S4: Rates of ACEs, obesity, and resilience, by special health-care needs (SHCN) status:**

|  |  | **SHCN** | **Non-SHCN** |
| --- | --- | --- | --- |
| **N** (weighted sample size) | | 7,174,894 | 22,848,533 |
| **Number of ACEs** (median [IQR]) | | 1 [0, 2] | 0 [0, 1] |
| **Family resilience score** (median [IQR]) | | 9.00 [8.00, 12.00] | 10 [8, 12] |
| **Weight status** (%) | |  |  |
|  | Normal/under | 65.3 | 70.6 |
|  | Overweight | 15 | 15.4 |
|  | Obese | 19.7 | 14.1 |

**Figure S3: Percent of children experiencing individual ACEs, by special health-care needs status:**


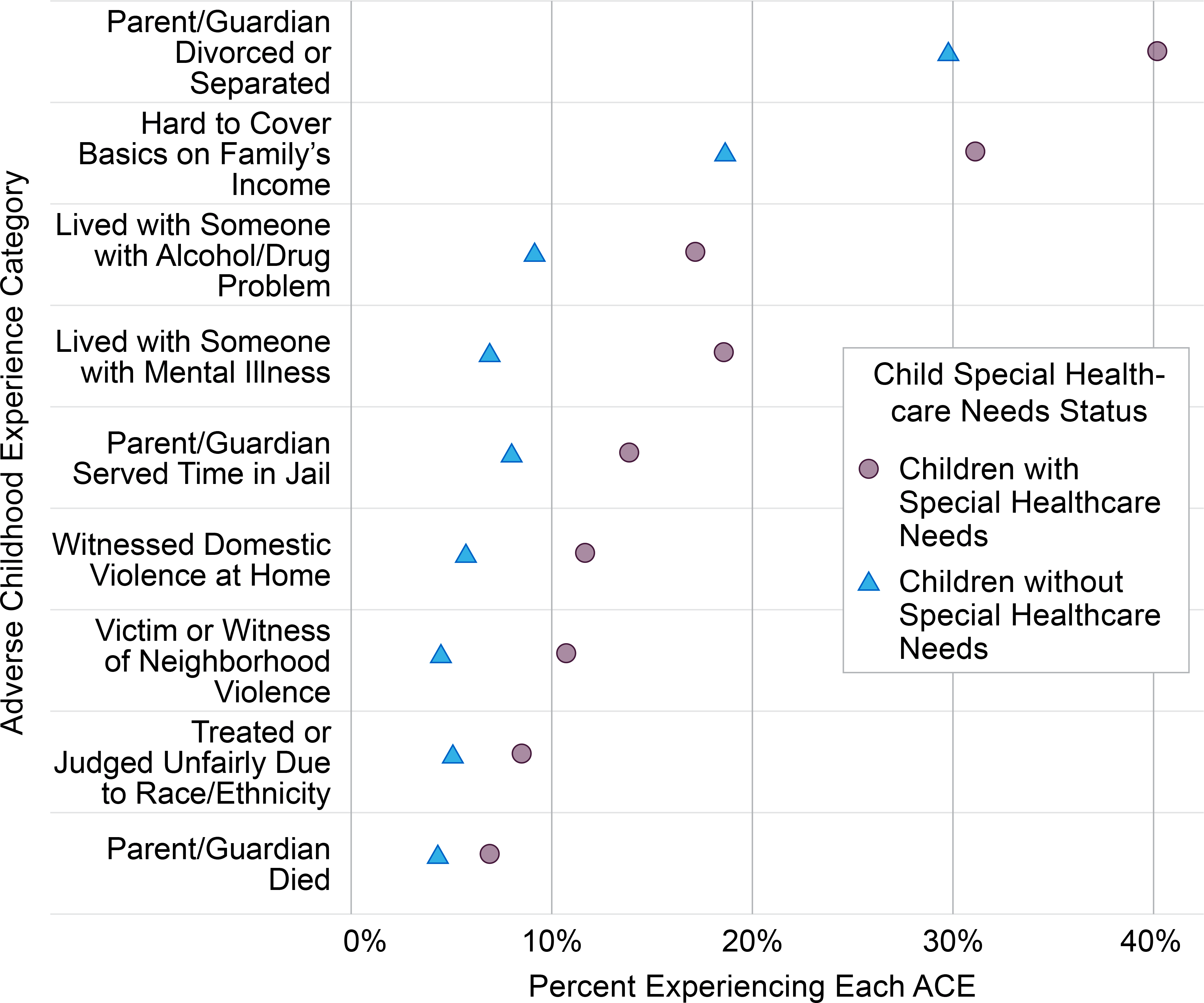


**Table S5: Model output for adjusted proportional odds regression model.**

Outcome: Child weight status (obesity, overweight, or normal/underweight)

Predictors of Interest: ACE count, Family Resilience, and their interaction

|  | **Estimate with 95% confidence interval** | **P-value** |
| --- | --- | --- |
| Exposure to 1 ACE | 1.94 (1.08, 3.49) | 0.027 |
| Exposure to 2–3 ACEs | 1.76 (1.06, 2.91) | 0.029 |
| Exposure to 4–9 ACEs | 1.87 (1.07, 3.26) | 0.027 |
| Family resilience score | 1.01 (0.97, 1.04) | 0.736 |
| Family resilience score X Exposure to 1 ACE | 0.96 (0.90, 1.02) | 0.157 |
| Family resilience score X Exposure to 2–3 ACEs | 0.98 (0.93, 1.03) | 0.474 |
| Family resilience score X Exposure to 4–9 ACEs | 0.97 (0.92, 1.03) | 0.355 |
| Child age | 0.93 (0.91, 0.95) | 0.000 |
| Child sex: female | 0.85 (0.77, 0.93) | 0.000 |
| Child race/ethnicity: Hispanic | 1.48 (1.30, 1.70) | 0.000 |
| Child race/ethnicity: Black, non-Hispanic | 1.57 (1.35, 1.83) | 0.000 |
| Child race/ethnicity: Other/Multi-racial, non-Hispanic | 1.09 (0.95, 1.26) | 0.203 |
| Child current health insurance: Yes | 1.14 (0.91, 1.44) | 0.254 |
| Child special healthcare needs: No | 0.82 (0.74, 0.91) | 0.000 |
| Adult highest education: High school or GED | 0.96 (0.76, 1.21) | 0.744 |
| Adult highest education: Some college or technical school | 0.81 (0.65, 1.03) | 0.083 |
| Adult highest education: College degree or higher | 0.49 (0.39, 0.62) | 0.000 |
| Family structure: Two parents, not currently married | 0.97 (0.78, 1.19) | 0.748 |
| Family structure: Single mother | 1.09 (0.93, 1.29) | 0.269 |
| Family structure: Other family type | 1.02 (0.84, 1.24) | 0.855 |
| Number of people in household: 3 | 1.17 (0.97, 1.42) | 0.097 |
| Number of people in household: 4 | 1.04 (0.86, 1.26) | 0.707 |
| Number of people in household: 5 | 0.87 (0.70, 1.07) | 0.183 |
| Number of people in household: 6 or more | 0.91 (0.73, 1.12) | 0.370 |
| Supportive neighborhood: No | 1.01 (0.91, 1.12) | 0.845 |
| Neighborhood amenities: 1 amenity | 1.05 (0.88, 1.26) | 0.565 |
| Neighborhood amenities: 2 amenities | 0.87 (0.74, 1.03) | 0.096 |
| Neighborhood amenities: 3 amenities | 0.88 (0.75, 1.02) | 0.087 |
| Neighborhood amenities: all 4 amenities | 0.83 (0.72, 0.96) | 0.010 |
| Intercept for Normal/under\|Overweight | 0.77 (0.43, 1.38) | 0.380 |
| Intercept for Overweight\|Obese | 1.95 (1.84, 2.07) | 0.000 |
